# Supplementary material for: Stratification of viral shedding patterns in saliva of COVID-19 patients
Source: eLife. 2026 Jan 16;13:RP96032. doi: 10.7554/eLife.96032 (PMC12810952; doi:10.7554/eLife.96032)
Supplement: Supplementary file 1. [file elife-96032-supp1.pdf]

Supplementary File 1

Stratification of viral shedding patterns in saliva of COVID-19 patients

Hyeongki Park[1,2], Raiki Yoshimura[1], Shoya Iwanami[1], Kwang Su Kim[1,3,4], Keisuke Ejima[5,6], Naotoshi Nakamura[1], Kazuyuki Aihara[7], Yoshitsugu Miyazaki[8], Takashi Umeyama[8], Ken Miyazawa[8], Takeshi Morita[9], Koichi Watashi[9], Christopher B. Brooke[10,11], Ruian Ke[12], Shingo Iwami[1,7, 13,14,15,16,17,†,*] and Taiga Miyazaki[18,†,*]

[1]interdisciplinary Biology Laboratory (iBLab), Division of Natural Science, Graduate School of Science, Nagoya University, Nagoya, Japan. [2]School of Biomedical Convergence Engineering, Pusan National University, Yangsan, South Korea. [3]Department of Science System Simulation, Pukyong National University, Busan, South Korea. [4]Department of Mathematics, Pusan National University, Busan, South Korea. [5]Lee Kong Chian School of Medicine, Nanyang Technological University, Singapore, Singapore. [6]The Tokyo Foundation for Policy Research, Tokyo, Japan. [7]International Research Center for Neurointelligence, The University of Tokyo Institutes for Advanced Study, The University of Tokyo, Tokyo, Japan. [8]Department of Chemotherapy and Mycoses, National Institute of Infectious Diseases, Tokyo, Japan. [9]Research Center for Drug and Vaccine Development, National Institute of Infectious Diseases, Tokyo, Japan. [10]Department of Microbiology, University of Illinois at Urbana-Champaign, Urbana, IL, USA. [11]Department of Statistics, University of Illinois at Urbana-Champaign, Urbana, IL, USA. [12]Theoretical Biology and Biophysics, Los Alamos National Laboratory, Los Alamos, NM, USA. [13]Institute of Mathematics for Industry, Kyushu University, Fukuoka, Japan. [14]Institute for the Advanced Study of Human Biology (ASHBi), Kyoto University, Kyoto, Japan. [15]Interdisciplinary Theoretical and Mathematical Sciences Program (iTHEMS), RIKEN, Saitama, Japan. [16]NEXT-Ganken Program, Japanese Foundation for Cancer Research (JFCR), Tokyo, Japan. [17]Science Groove Inc., Fukuoka, Japan. [18]Division of Respirology, Rheumatology, Infectious Diseases, and Neurology, Department of Internal Medicine, Faculty of Medicine, University of Miyazaki, Miyazaki, Japan.

Table A | Daily symptom data for whole cohorts for each group

Daily symptom	Group1 (N=46)	Group2 (N=61)	Group3 (N=37)	Overall (N=144)
Cough	84.8%	75.4%	64.9%	75.7%
Dyspnea	32.6%	24.6%	37.8%	30.6%
Nasal discharge	58.7%	67.2%	59.5%	62.5%
Sore throat	63.0%	65.6%	59.5%	63.2%
Nausea/vomiting/diarrhea	52.2%	68.9%	54.1%	59.7%
Myalgia	39.1%	57.4%	45.9%	48.6%
Olfaction abnormal	41.3%	52.5%	59.5%	50.7%
Fever	45.7%	24.6%	32.4%	33.3%

Table B | Summary of parameter estimation by the model described in Eqs.(1-2)

Parameters	Description	Unit	$\boldsymbol{\vartheta}$: Fixed effect (SE)*	$\boldsymbol{\Omega}$: SD of random effect (SE)*
β	Rate constant for virus infection	$(\text{RNA copies/ml})^{-1}\text{day}^{-1}$	2.08×10^{-7} (6.79×10^{-8})	2.79 (0.26)
γ	Maximum rate constant for viral replication	Day^{-1}	13.2 (1.44)	0.45 (0.08)
δ	Death rate of virus-producing cells	Day^{-1}	0.97 (0.05)	0.54 (0.05)
τ	Days from infection to symptom onset	Days	3.73 (0.21)	0.31 (0.05)

* The parameter for patient k, ϑ_i $(= \vartheta \times e^{\pi_k})$ is represented as a product of ϑ (a fixed effect) and e^{π_k} (a random effect). π_k follows the normal distribution with mean 0 and standard deviation Ω. SE: standard error.

Table C | Summary of parameter estimation by the model described in Eqs.(3-6)

Parameters	Description	Unit	ϑ: Fixed effect (SE)*	Ω: SD of random effect (SE)*
β	Rate constant for virus infection	(RNA copies/ml)$^{-1}$day^{-1}	7.74×10^{-6} (2.59×10^{-6})	2.04 (0.27)
δ_1	Death rate of virus-producing cells	Day^{-1}	0.58 (0.05)	0.52 (0.06)
δ_2	Death rate of virus-producing cells by immune effector cells	Day^{-1}	1.09 (0.13)	0.6 (0.11)
t_1	Days from symptom onset when immune effector cells start to work	Days	10.5 (2.61)	1.44 (0.24)
π	Rate constant for virus production	Day^{-1}	1.73 (0.59)	2.61 (0.31)
τ	Days from infection to symptom onset	Days	4.13 (0.28)	0.44 (0.06)

* The parameter for patient k, ϑ_i $(= \vartheta \times e^{\pi_k})$ is represented as a product of ϑ (a fixed effect) and e^{π_k} (a random effect). π_k follows the normal distribution with mean 0 and standard deviation Ω. SE: standard error.

Table D | Features of reconstructed individual viral dynamics for each group

Feature	Group1 (N=46)	Group2 (N=61)	Group3 (N=37)	Overall (N=144)	p-value
Duration of virus shedding (days)	11.5 (3.16)*	17.4 (3.14)	30.0 (5.8)	18.8 (8.13)	<0.001
Peak viral load (RNA copies/ml)	6.94 (0.69)	8.21 (0.56)	7.95 (1.03)	7.74 (0.93)	0.020
Up-slope (day^{-1})	12.2 (1.65)	11.2 (1.85)	13 (1.18)	12 (1.79)	<0.001
Down-slope (day^{-1})	1.53 (0.55)	1.11 (0.24)	0.57 (0.13)	1.1 (0.51)	<0.001

* Mean (standard deviation)

Table E | Pearson's correlation coefficients between clinical data and features of viral dynamics.

Clinical data	Duration of viral RNA detection		Peak viral load		Up-slope		Down-slope	
	Pearson's r	p-value	Pearson's r	p-value	Pearson's r	p-value	Pearson's r	p-value
Age	-0.08	0.8359	-0.16	0.5783	0.15	0.5880	0.08	0.8141
Systolic blood pressure	0.08	0.8359	-0.04	0.9388	0.12	0.6448	-0.16	0.4519
Diastolic blood pressure	-0.06	0.8359	-0.2	0.5152	0.14	0.5880	-0.03	0.9204
Pulse rate	-0.13	0.6307	-0.05	0.9388	0	0.9710	0.01	0.9445
SpO_2	-0.04	0.8359	-0.06	0.9388	0.07	0.9178	0.01	0.9512
Respiratory rate	0	0.9770	0.02	0.9765	0.08	0.9178	0.04	0.8681
White blood cell count	-0.07	0.8359	0	0.9868	-0.01	0.9710	0.07	0.8610
Neutrophil	-0.04	0.8359	-0.02	0.9765	0.15	0.5880	0.05	0.8681
Eosinophil	-0.04	0.8359	-0.24	0.4083	0.16	0.5880	-0.08	0.8386
Basophil	0.09	0.8359	-0.05	0.9388	-0.15	0.5880	-0.14	0.4933
Lymphocytes	0.01	0.9770	0.04	0.9388	-0.15	0.5880	-0.02	0.9231
Monocyte	0.11	0.6993	0.08	0.8939	-0.09	0.9166	-0.06	0.8681
Red blood cell count	0.08	0.8359	0.07	0.8939	0.01	0.9710	0.01	0.9445
Amount of hemoglobin	0.26	0.1688	0.26	0.4083	0	0.9710	-0.2	0.3194
Hematocrit	0.25	0.1688	0.18	0.5209	0.07	0.9178	-0.2	0.3194
Platelet count	-0.05	0.8359	-0.13	0.6790	0.03	0.9710	-0.04	0.8681
CRP	-0.06	0.8359	0.01	0.9765	0.02	0.9710	0.13	0.4933
Protein	0	0.9770	-0.1	0.8684	0.08	0.9178	-0.03	0.9200
Albumin	0.22	0.1688	0.21	0.5152	-0.15	0.5880	-0.17	0.3756
ALT	0.24	0.1688	0.04	0.9388	0.14	0.5880	-0.24	0.2570
AST	0.26	0.1688	0.16	0.5783	0.1	0.8732	-0.23	0.2570
γ-GTP	0.16	0.4469	-0.07	0.8939	0.18	0.5880	-0.21	0.3194
ALP	0.21	0.1791	-0.07	0.8939	0.26	0.4708	-0.27	0.2570
LDH	0.12	0.6676	-0.01	0.9765	0.06	0.9178	-0.15	0.4661
Bilirubin	0.22	0.1688	0.15	0.5783	0.03	0.9710	-0.19	0.3194
CK	0.08	0.8359	0.01	0.9765	-0.04	0.9710	-0.12	0.4971
Na	-0.02	0.9374	-0.12	0.7050	0.02	0.9710	-0.05	0.8681
K	-0.02	0.9374	-0.08	0.8939	0.06	0.9178	0.07	0.8610
Cl	-0.07	0.8359	-0.19	0.5209	0.06	0.9178	-0.04	0.8681
BUN	0.22	0.1688	-0.03	0.9388	0.13	0.6448	-0.25	0.2570
CRE	0.22	0.1688	0.18	0.5209	0.03	0.9710	-0.14	0.4933
Blood glucose	0.04	0.8359	-0.12	0.7050	0.15	0.5880	-0.06	0.8681
HbA1c	-0.04	0.8359	-0.13	0.6790	0.07	0.9178	0.04	0.8681
Procalcitonin	-0.14	0.5327	0.05	0.9388	-0.18	0.5880	0.19	0.3194

Fibrinogen	-0.08	0.8359	0.02	0.9765	0.04	0.9710	0.13	0.4933
PT	0.16	0.4469	0.03	0.9388	-0.02	0.9710	-0.12	0.4971
APTT	-0.05	0.8359	0.08	0.8939	0.01	0.9710	0.13	0.4971
PT-INR	-0.22	0.1688	-0.01	0.9765	-0.01	0.9710	0.18	0.3655
D-dimer	-0.06	0.8359	-0.13	0.6790	0.13	0.6448	-0.01	0.9512

Table F | Salivary micro-RNAs obtained from 60 specimens of 30 participants

Micro-RNA	Group1 (N=10)	Group2 (N=10)	Group3 (N=10)	Overall (N=30)
mir-10	45.4 (28.98)	27.5 (11.41)	34.25 (23.71)	35.72 (23.43)
mir-103	0.12 (0.27)	0.42 (0.85)	0.11 (0.23)	0.22 (0.54)
mir-1180	2.16 (4.39)	0.94 (1.3)	0.81 (1.45)	1.3 (2.79)
mir-1226	0.85 (1.3)	0.63 (0.85)	0.44 (0.74)	0.64 (0.99)
mir-1253	0.04 (0.16)	0 (0)	0 (0)	0.01 (0.09)
mir-1255	0.09 (0.23)	0 (0)	0 (0)	0.03 (0.14)
mir-128	0 (0)	0.03 (0.12)	0.01 (0.05)	0.01 (0.07)
mir-1296	1.25 (2.41)	0.41 (0.63)	0.46 (0.78)	0.71 (1.53)
mir-130	1.02 (1.98)	0.74 (0.91)	1.04 (1.12)	0.93 (1.4)
mir-145	0 (0)	0.01 (0.06)	0.02 (0.1)	0.01 (0.07)
mir-146	9.72 (4.52)	11.41 (6.49)	10.98 (6.22)	10.71 (5.76)
mir-148	28.7 (5.9)	31.47 (6.88)	29.53 (10.38)	29.9 (7.91)
mir-154	0.6 (1.95)	1 (1.68)	0.82 (1.88)	0.81 (1.82)
mir-16	174.2 (34.41)	178.55 (46.78)	184.2 (37.33)	178.98 (39.39)
mir-17	0 (0)	0.02 (0.11)	0 (0)	0.01 (0.06)
mir-181	0.93 (1.36)	0.99 (1.1)	1.28 (2.15)	1.07 (1.58)
mir-185	7.39 (7.39)	5.04 (2.31)	7.87 (13.71)	6.77 (9.02)
mir-19	14.13 (8.99)	17.23 (12.16)	11.49 (6.35)	14.28 (9.6)
mir-191	2.87 (5.2)	1.22 (1.54)	1.51 (1.68)	1.87 (3.3)
mir-192	0.14 (0.46)	0.07 (0.3)	0.04 (0.13)	0.08 (0.32)
mir-193	0.16 (0.7)	0 (0)	0 (0)	0.05 (0.4)
mir-204	0.21 (0.6)	0.61 (1.66)	0.18 (0.41)	0.33 (1.05)
mir-210	0.41 (1.5)	1.49 (3.46)	0.14 (0.4)	0.68 (2.23)
mir-214	0 (0)	0 (0)	0.01 (0.05)	0 (0.03)
mir-218	0.03 (0.15)	0 (0)	0 (0)	0.01 (0.09)
mir-224	0 (0)	0 (0)	0.04 (0.17)	0.01 (0.1)
mir-23	0.42 (0.97)	0.26 (0.6)	0.22 (0.41)	0.3 (0.69)
mir-24	0.21 (0.44)	0.13 (0.3)	0.1 (0.22)	0.15 (0.33)
mir-26	33.95 (15.04)	35.26 (13.11)	32.99 (7.73)	34.07 (12.18)
mir-28	8.2 (3.78)	7.68 (4.77)	10.76 (23.76)	8.88 (13.99)
mir-29	5.66 (3.95)	5.51 (2.56)	4.93 (2.81)	5.37 (3.12)
mir-290	0 (0)	0 (0)	0.03 (0.14)	0.01 (0.08)
mir-296	0 (0)	0.11 (0.49)	0 (0)	0.04 (0.28)
mir-3180	0.02 (0.11)	0 (0)	0.06 (0.28)	0.03 (0.17)

mir-320	0.74 (1.15)	0.47 (0.78)	0.82 (1.34)	0.68 (1.11)
mir-33	0.01 (0.06)	0.04 (0.17)	0.02 (0.1)	0.02 (0.12)
mir-331	5.34 (5.31)	4.07 (3.57)	3.35 (3.53)	4.25 (4.23)
mir-338	1.45 (1.66)	1.55 (2.17)	1.07 (1.21)	1.36 (1.71)
mir-34	0.51 (1.77)	0.49 (1.31)	1.83 (3.75)	0.94 (2.55)
mir-340	0.01 (0.05)	0 (0)	0 (0)	0 (0.03)
mir-342	0 (0)	0.02 (0.1)	0 (0)	0.01 (0.06)
mir-345	6.98 (7.32)	4.14 (3.53)	5.2 (5.86)	5.44 (5.81)
mir-365	0.44 (1.5)	0.33 (0.6)	0.35 (0.95)	0.37 (1.06)
mir-375	0.14 (0.32)	0.18 (0.38)	1.03 (3.51)	0.45 (2.06)
mir-425	6.94 (2.85)	5.84 (2.51)	7 (4.82)	6.59 (3.52)
mir-449	0.07 (0.29)	0.04 (0.12)	0.77 (2.29)	0.29 (1.36)
mir-489	0 (0)	0.03 (0.15)	0 (0)	0.01 (0.09)
mir-491	0 (0)	0.13 (0.58)	0 (0)	0.04 (0.34)
mir-492	0 (0)	0 (0)	1.59 (7.1)	0.53 (4.1)
mir-500	2.22 (2.69)	2.12 (1.59)	1.79 (1.9)	2.04 (2.08)
mir-505	3.22 (2.83)	2.68 (3.79)	2.91 (3.43)	2.94 (3.32)
mir-544	0.3 (1.34)	0.08 (0.25)	0.19 (0.68)	0.19 (0.87)
mir-548	0.32 (0.69)	0.31 (0.86)	0.81 (2.26)	0.48 (1.45)
mir-550	0.43 (1.1)	0.61 (1.47)	0.36 (0.84)	0.46 (1.15)
mir-551	0.01 (0.05)	0.05 (0.17)	0 (0)	0.02 (0.1)
mir-552	0.01 (0.06)	0 (0)	0 (0)	0 (0.04)
mir-562	0.05 (0.16)	0 (0)	0.6 (2.68)	0.22 (1.55)
mir-572	0 (0)	0.05 (0.23)	0 (0)	0.02 (0.13)
mir-574	20.93 (14.57)	15.29 (10.63)	16.91 (15.11)	17.71 (13.56)
mir-576	2.81 (5.95)	1.47 (1.5)	2.71 (4.5)	2.33 (4.36)
mir-581	0 (0)	0.01 (0.06)	0.04 (0.17)	0.02 (0.1)
mir-582	0.23 (0.45)	0.8 (1.24)	0.31 (0.41)	0.45 (0.82)
mir-584	1.27 (1.63)	2.77 (6.44)	2.41 (2.84)	2.15 (4.15)
mir-589	0.37 (0.73)	0.37 (0.68)	0.3 (0.52)	0.35 (0.64)
mir-597	0.04 (0.12)	0.08 (0.3)	0.19 (0.6)	0.1 (0.39)
mir-598	14.96 (15.14)	7.66 (6.68)	11.74 (10.09)	11.46 (11.4)
mir-612	0.03 (0.13)	0.02 (0.08)	0 (0)	0.02 (0.09)
mir-616	0.05 (0.17)	0.2 (0.4)	0.16 (0.41)	0.14 (0.34)
mir-618	1.2 (1.61)	1.32 (1.54)	0.66 (0.95)	1.06 (1.41)
mir-624	0.19 (0.39)	0.52 (0.95)	0.27 (0.5)	0.33 (0.67)
mir-628	0.9 (1.29)	2.33 (3.55)	0.67 (0.77)	1.3 (2.31)
mir-632	0 (0)	0.05 (0.23)	0.01 (0.06)	0.02 (0.14)
mir-636	0 (0)	0.08 (0.36)	0.03 (0.15)	0.04 (0.23)

mir-639	0.1 (0.46)	0 (0)	0 (0)	0.03 (0.26)
mir-642	1.05 (1.88)	1.27 (2.29)	0.46 (0.61)	0.93 (1.75)
mir-643	0.06 (0.25)	0.1 (0.27)	0.01 (0.06)	0.06 (0.21)
mir-650	0 (0)	0.23 (0.91)	0 (0)	0.08 (0.53)
mir-651	0.39 (1.08)	0.39 (1.31)	0.15 (0.33)	0.31 (0.99)
mir-671	2.89 (3.29)	1.47 (1.67)	1.71 (3.6)	2.02 (2.99)
mir-672	0 (0)	0.04 (0.2)	0 (0)	0.01 (0.12)
mir-675	0.01 (0.06)	0 (0)	0 (0)	0 (0.03)
mir-692	0.01 (0.06)	0.16 (0.4)	0.3 (1.34)	0.16 (0.8)
mir-720	0 (0)	0.02 (0.1)	3.17 (14.19)	1.07 (8.19)
mir-744	18.41 (20.9)	13.73 (12.2)	14.16 (16.82)	15.43 (16.86)
mir-765	0.13 (0.46)	0.09 (0.36)	0.17 (0.33)	0.13 (0.38)
mir-885	1.23 (1.96)	1.15 (2.34)	0.72 (0.98)	1.03 (1.83)
mir-887	0.02 (0.11)	0.02 (0.1)	0.02 (0.08)	0.02 (0.1)
mir-938	0 (0)	0 (0)	0.05 (0.19)	0.02 (0.11)
mir-941	13.61 (10.91)	7.37 (4.13)	7.17 (4.16)	9.38 (7.65)
mir-944	0.19 (0.45)	0.6 (0.96)	0.91 (1.27)	0.57 (0.99)
mir-1846	4.96 (6.31)	1.67 (5.15)	11.6 (27.8)	6.08 (16.96)
mir-811	18.1 (17.8)	12.64 (23.67)	39.74 (78.61)	23.49 (49.11)

* mean (standard deviation)

Table G | Spearman's correlation coefficients between micro-RNA data and features of viral dynamics.

Micro-RNA	Duration of viral shedding		Peak viral load		Up-slope		Down-slope	
	Spearman's ρ	p-value	Spearman's ρ	p-value	Spearman's ρ	p-value	Spearman's ρ	p-value
mir-10	-0.16	0.9507	-0.31	0.2323	0.06	0.944	0.07	0.9867
mir-103	0.14	0.9507	0.06	0.8198	0.07	0.9321	-0.16	0.9867
mir-1180	-0.08	0.9982	0.2	0.597	-0.16	0.8616	0.12	0.9867
mir-1226	-0.03	0.9982	0.09	0.7441	0	0.993	0.03	0.9867
mir-1253	-0.16	0.9507	0.02	0.9244	-0.17	0.8616	0.17	0.9867
mir-1255	-0.33	0.6991	-0.09	0.7441	-0.08	0.9247	0.34	0.7121
mir-128	0.04	0.9982	0.12	0.6894	-0.09	0.9247	-0.01	0.9867
mir-1296	-0.01	0.9982	0.17	0.6225	0.02	0.9497	0.02	0.9867
mir-130	0.13	0.9507	0.18	0.597	0.02	0.9497	-0.07	0.9867
mir-145	0.12	0.9553	0.01	0.9822	0.08	0.9247	-0.12	0.9867
mir-146	0.17	0.9507	0.16	0.635	0.09	0.9247	-0.15	0.9867
mir-148	-0.02	0.9982	0.25	0.4697	-0.11	0.9029	0.04	0.9867
mir-154	-0.03	0.9982	0.14	0.635	-0.18	0.8616	0.07	0.9867
mir-16	0.25	0.7309	0.07	0.7731	0.18	0.8616	-0.27	0.9509
mir-17	0.02	0.9982	0.04	0.87	-0.04	0.9497	-0.04	0.9867
mir-181	0.08	0.9982	0.06	0.8198	0	0.9925	-0.07	0.9867
mir-185	-0.07	0.9982	-0.19	0.597	0.08	0.9247	0	0.9918
mir-19	-0.09	0.9982	0.25	0.4697	-0.16	0.8616	0.16	0.9867
mir-191	-0.01	0.9982	-0.14	0.635	0.12	0.9029	-0.04	0.9867
mir-192	-0.03	0.9982	-0.06	0.8198	0.19	0.8616	0.01	0.9867
mir-193	-0.17	0.9507	-0.1	0.7441	0.02	0.9497	0.16	0.9867
mir-204	-0.02	0.9982	0.28	0.3167	-0.25	0.8616	0.06	0.9867
mir-210	-0.04	0.9982	0.21	0.597	-0.21	0.8616	0.06	0.9867
mir-214	0.14	0.9507	-0.02	0.9244	0.14	0.9029	-0.13	0.9867
mir-218	-0.16	0.9507	0.02	0.9244	-0.17	0.8616	0.17	0.9867
mir-224	0.22	0.8213	0.16	0.635	0.22	0.8616	-0.2	0.9867
mir-23	-0.02	0.9982	0.08	0.7719	0.11	0.9029	0.03	0.9867
mir-24	-0.1	0.9982	-0.08	0.7731	0.05	0.944	0.11	0.9867
mir-26	0.07	0.9982	-0.06	0.8198	0.01	0.9736	-0.09	0.9867
mir-28	-0.27	0.6991	-0.11	0.6894	-0.09	0.9247	0.23	0.9867
mir-29	-0.07	0.9982	0	0.9926	-0.12	0.9029	0.06	0.9867
mir-290	0.2	0.9175	0.11	0.6894	0.19	0.8616	-0.17	0.9867
mir-296	-0.02	0.9982	0.08	0.7731	-0.15	0.8892	0.04	0.9867
mir-3180	-0.02	0.9982	-0.2	0.597	0.12	0.9029	-0.01	0.9867

mir-320	-0.01	0.9982	-0.12	0.6615	-0.03	0.9497	0	0.9979
mir-33	0	0.9982	-0.05	0.8198	0.13	0.9029	0.01	0.9867
mir-331	-0.03	0.9982	-0.05	0.8373	-0.05	0.944	-0.01	0.9867
mir-338	0	0.9982	0.16	0.635	0.08	0.9247	0.02	0.9867
mir-34	0.27	0.6991	0.12	0.6615	0.12	0.9029	-0.24	0.9867
mir-340	-0.13	0.9507	-0.11	0.6894	0.05	0.944	0.11	0.9867
mir-342	0.01	0.9982	0.2	0.597	-0.18	0.8616	0.01	0.9867
mir-345	-0.15	0.9507	-0.13	0.6517	-0.08	0.9247	0.11	0.9867
mir-365	0.06	0.9982	0	0.9926	0.09	0.9247	-0.09	0.9867
mir-375	0.13	0.9507	0.05	0.8198	0.07	0.9321	-0.09	0.9867
mir-425	-0.11	0.985	-0.14	0.635	0.01	0.9724	0.06	0.9867
mir-449	0.29	0.6991	0.14	0.635	0.17	0.8616	-0.27	0.9509
mir-489	-0.11	0.985	0.09	0.7441	-0.2	0.8616	0.14	0.9867
mir-491	0.07	0.9982	0.14	0.635	-0.08	0.9247	-0.07	0.9867
mir-492	0.16	0.9507	-0.01	0.9867	0.17	0.8616	-0.14	0.9867
mir-500	-0.03	0.9982	0.16	0.635	-0.08	0.9247	0.07	0.9867
mir-505	0	0.9982	-0.22	0.597	0.16	0.8616	-0.07	0.9867
mir-544	0.11	0.985	-0.05	0.8198	-0.02	0.9497	-0.13	0.9867
mir-548	0.03	0.9982	-0.11	0.6894	0.02	0.9497	-0.04	0.9867
mir-550	-0.1	0.9982	-0.02	0.9244	-0.03	0.9497	0.14	0.9867
mir-551	-0.05	0.9982	0.17	0.6225	-0.17	0.8616	0.06	0.9867
mir-552	-0.09	0.9982	-0.07	0.8012	-0.02	0.9497	0.09	0.9867
mir-562	-0.09	0.9982	-0.17	0.6207	-0.08	0.9247	0.06	0.9867
mir-572	0.04	0.9982	0.13	0.6605	-0.1	0.9247	-0.02	0.9867
mir-574	-0.14	0.9507	-0.18	0.597	-0.02	0.9497	0.08	0.9867
mir-576	0	0.9982	0.09	0.7511	-0.07	0.9331	0.02	0.9867
mir-581	0.17	0.9507	0.14	0.635	0.13	0.9029	-0.17	0.9867
mir-582	0.07	0.9982	0.2	0.597	-0.17	0.8616	-0.01	0.9867
mir-584	0.15	0.9507	0.04	0.8503	-0.07	0.9331	-0.16	0.9867
mir-589	0.06	0.9982	0.13	0.6517	0.06	0.944	-0.03	0.9867
mir-597	-0.03	0.9982	-0.1	0.724	0.05	0.944	0.03	0.9867
mir-598	-0.08	0.9982	-0.32	0.2323	0.11	0.9029	0	0.9918
mir-612	-0.07	0.9982	0.1	0.724	-0.17	0.8616	0.1	0.9867
mir-616	0.13	0.9507	0.19	0.597	-0.07	0.936	-0.08	0.9867
mir-618	-0.13	0.9507	0.02	0.9353	-0.05	0.944	0.13	0.9867
mir-624	0.03	0.9982	0.18	0.597	-0.13	0.9029	0.01	0.9867
mir-628	0.03	0.9982	0.18	0.597	-0.08	0.9247	0.01	0.9867
mir-632	0.08	0.9982	0.29	0.3167	-0.13	0.9029	-0.02	0.9867
mir-636	0.15	0.9507	0.09	0.7511	0.04	0.9497	-0.14	0.9867

mir-639	-0.06	0.9982	-0.19	0.597	0.2	0.8616	-0.01	0.9867
mir-642	-0.02	0.9982	0.09	0.7511	0.05	0.944	0.03	0.9867
mir-643	-0.02	0.9982	0.24	0.4955	-0.17	0.8616	0.05	0.9867
mir-650	-0.01	0.9982	0.07	0.8012	-0.12	0.9029	0.03	0.9867
mir-651	-0.01	0.9982	0.09	0.7511	-0.04	0.9497	0.05	0.9867
mir-671	-0.13	0.9507	-0.05	0.8198	0.04	0.944	0.1	0.9867
mir-672	0.01	0.9982	0.2	0.597	-0.18	0.8616	0.01	0.9867
mir-675	-0.22	0.8213	-0.14	0.635	0.1	0.9247	0.22	0.9867
mir-692	-0.03	0.9982	0.05	0.8198	-0.2	0.8616	0.05	0.9867
mir-720	0.12	0.9553	0.14	0.635	0	0.993	-0.1	0.9867
mir-744	-0.05	0.9982	-0.2	0.597	0.06	0.944	-0.03	0.9867
mir-765	0.25	0.7309	-0.08	0.7559	0.38	0.2349	-0.26	0.9509
mir-885	-0.07	0.9982	0	0.9926	0.01	0.9724	0.09	0.9867
mir-887	0.09	0.9982	0.14	0.635	0.03	0.9497	-0.06	0.9867
mir-938	0.22	0.8213	0.14	0.635	0.14	0.9029	-0.17	0.9867
mir-941	-0.22	0.8213	-0.37	0.1235	0.12	0.9029	0.08	0.9867
mir-944	0.28	0.6991	0.37	0.1235	-0.01	0.9925	-0.2	0.9867
mir-1846	-0.17	0.9507	-0.53	0.0011	0.2	0.8616	0.09	0.9867
mir-811	-0.13	0.9507	-0.31	0.2323	0.05	0.944	0.06	0.9867
